# Gender Differences in Sports-Related Child Maltreatment: A Systematic Review

**DOI:** 10.1177/15248380251372157

**Published:** 2025-09-27

**Authors:** Annie Yun An Shiau, Kayla D. Schick, Kelly McWilliams, Shanna Dewit-Williams

**Affiliations:** 1 McGill University, Montreal, QC, Canada; 2 CUNY, New York, NY, USA

**Keywords:** child maltreatment, sports, gender differences, child athletes, violence

## Abstract

Sports-related child maltreatment (CM) has emerged as a global issue in the past few decades. Despite evidence pointing to gender differences in how children experience and report CM, this literature remains fragmented and limited to sexual abuse in sports. This systematic review identified and synthesized available evidence on the prevalence, perpetrators, outcomes, and disclosures of various sports-related CM subtypes to determine whether these experiences differ across genders. Systematic searches were conducted using three databases (i.e., PsycInfo, MedLine, Scopus) for studies published between the earliest date of each database to May 2024. A total of 509 unique articles were returned, from which 22 met the inclusion criteria. The included studies examined children or adults who participated in organized sports before age 18 and compared a quantitative measure of sports-related CM across genders. Findings revealed higher rates of sports-related neglect, psychological, and sexual abuse reported by girls, and higher rates of sports-related physical abuse reported by boys. Gender differences were also observed in the common perpetrators and disclosure recipients, with boys reporting more peer-perpetrated violence and disclosures made to coaches, and girls reporting more coach-perpetrated violence and disclosures made to parents. Regardless, severe outcomes of sports-related CM and concerns of underreporting were shared across genders. Future research is needed to establish consistent measurement tools and to explore gender differences in the perceptions and barriers to disclosing all forms of sports-related CM. The need to develop formal reporting structures in sports is also highlighted for policymakers and practitioners.

Today, over half of the children in the U.S. participate in sports (Black et al., 2022) for its numerous mental, physical, and social benefits (Eime et al., 2013). However, the risk of violence against children in sports has emerged as a global and serious issue within the past three decades (Parent & Fortier, 2018). Researchers began to warn against sports-related child maltreatment (CM) in the 1990s (e.g., Brackenridge, 1994), and since then, a growing number of literature has examined its prevalence and consequences for young athletes (e.g., Parent & Fortier, 2018; Tuakli-Wosnornu et al., 2024). Disclosures have also been made by athletes in North America (e.g., Ward & Strashin, 2019) and around the world (e.g., Harrison, 2022), with high-profile cases (e.g., the Larry Nassar case) receiving substantial media attention and highlighting, to the public, the need to protect children in sports (Mountjoy, 2019).

## Child Maltreatment in Sports

The World Health Organization (2022) defines CM as an umbrella term that encompasses:

all types of physical and/or emotional ill-treatment, sexual abuse, neglect, negligence and commercial or other exploitation, which results in actual or potential harm to the child’s health, survival, development or dignity in the context of a relationship of responsibility, trust or power (para. 1).

Definitions and categorizations of CM can vary across legal and research contexts, including considerations around whether peer-perpetrated violence constitutes maltreatment. This review used the conceptual framework of maltreatment in sports proposed by Stirling (2009), where maltreatment is considered within relationships of differential power and can occur at the hands of coaches, parents, administrators, officials, and/or peers.

Researchers have identified various individual (e.g., sports level, race/ethnicity) and interpersonal (e.g., organisational climate) variables as risk factors for violence in sports in general (Tuakli-Wosornu et al., 2024). Several factors render the sports context conducive specifically to CM. Coaches hold a unique position of power due to the close nature of their relationship with child athletes and act as gatekeepers to the sport (e.g., selecting players and determining positions) (Parent & Demers, 2011). They are often trusted by parents and may even take on a parental role in athletes’ lives (Wilinsky & McCabe, 2021). This power dynamic and dependency make athletes especially vulnerable to coaches with the intention to abuse this relationship (Wilinsky & McCabe, 2021). The normalization of touch in sports is another factor that puts child athletes at risk, especially for sexual and physical abuse (McPherson et al., 2016). Not only is contact encouraged in certain sports, but research has documented that both athletes and coaches report that touch is necessary for instruction, injury assessment, and even positive affirmation (Kerr et al., 2015; McPherson et al., 2016). Young athletes may struggle to recognize or doubt themselves when a touch is no longer instructive and becomes either ambiguous or even seductive or harmful. Lastly, sports’ culture of control further exposes athletes to manipulation and violence. Funding for sports institutions often depends on athletes’ performance, and athletes are motivated by punishments (e.g., being traded or benched) if they do not perform (Kerr et al., 2020). Under a winning-at-all-costs mentality, athletes are susceptible to violence that they may not tolerate in other spaces (e.g., school), such as overtraining, grooming, being belittled, or having their weight and bodies strictly controlled, in order to succeed (Kerr et al., 2020; McPherson et al., 2016).

Not only are children vulnerable to CM in sports, but they also face additional barriers in disclosing sports-related abuse whether formally (e.g., police) and/or informally (e.g., parents, peers). They may fear losing their sports career, disbelief, or disinterest from their organization (Parent, 2011). Even for those who do decide to report, dead-end disclosures (i.e., disclosure recipients who did not further report) and a lack of administrative awareness of CM remain major concerns in sports (Parent, 2011). For instance, teammates who witnessed CM are silenced through threats of negative repercussions, not wanting to rock the boat if they wish to continue to be successful themselves (Kirby et al., 2002).

With increased public exposure and media attention, the number of empirical research examining sports-related CM has grown. In recent years, several reviews of literature have synthesized the prevalence (Parent & Fortier, 2017) and overall knowledge (McPherson et al., 2016) of CM in sports. Despite this, two significant gaps remain in this field: (a) the examination of gender differences, and (b) the examination of various CM subtypes.

## Gender Differences

Examination of CM outside of sports has highlighted important gender^1^ differences in the experiences and reporting of CM. In North America, for instance, rates of substantiated, adult-perpetrated sexual abuse, psychological abuse, and neglect were higher for girls, whereas rates of physical abuse were similar across genders (Moody et al., 2018). Consequences for CM were gendered as well, with maltreated boys displaying more externalizing behaviors (e.g., violent youth offending; Baidawi et al., 2023) while maltreated girls developing more internalizing problems (e.g., depression and anxiety; Gallo et al., 2018). Gender has also consistently been identified as an influential factor in CM disclosure, especially for child sexual abuse (CSA) (Alaggia et al., 2019). Not only are boys more reluctant to disclose all forms of CM (Lev-Wiesel & First, 2018), but they may be especially reluctant to disclose CSA due to fear of being viewed as homosexual and stigmatization (Alaggia et al., 2019). In comparison, girls are more troubled by feelings of guilt and fear of not being believed (Alaggia et al., 2019). Transgender and gender non-conforming children are further found to be at even higher risks of CM and its consequences compared to cisgender peers (e.g., Thoma et al., 2021). Thus, there are reasons to believe that gender differences exist and must be examined for sports-related CM as well. For instance, boys make up the majority of sports participants worldwide yet have been understudied in CSA literature in sports (Parent & Bannon, 2012). The restriction of gender to a binary concept in sports may also increase the risks of stigmatization and violence against transgender or gender non-conforming athletes. Understanding these differences will help determine whether and how approaches for preventing and supporting young athletes who experienced CM in sports should be differentiated by gender.

## Maltreatment Beyond Child Sexual Abuse

An overwhelming majority of CM research and media attention both in (Mountjoy, 2019; Parent & Fortier, 2018) and out of sports (Moody et al., 2018) has focused on CSA and child sexual harassment and abuse (SHA) (i.e., a continuum of sexual harassment, like repeated unwanted sexual remarks, to abuse, like rape; SHA). While CSA research in sports has identified several risk factors (e.g., self-sacrifice; Demers et al., 2021), it remains unclear whether these also put individuals at risk for other forms of CM. Further, whether those who do not fir CSA risk profiles are still vulnerable to other forms of sports-related CM remains underexplored. Other forms of sports-related CM, including physical abuse, psychological abuse, and neglect, exist and may even take on distinctive forms in sports. In addition to traditional forms of physical abuse (e.g., hitting), physical abuse in sports could take place between athletes during hazing or through intense training programs (e.g., training in extreme heat or when injured) (Parent & Fortier, 2018). Further, the winning-at-all-costs attitude in sports could lead to unique forms of psychological abuse, such as belittling or humiliating athletes’ performance, in pursuit of athletic success (Fortier et al., 2020). By constantly pushing themselves physically, athletes are also prone to neglect, including injuries and disordered eating under inadequate supervision (Fortier et al., 2020). CM beyond CSA all yield real and significant consequences for children in sports (Parent & Fortier, 2018). Thus, limiting empirical research to CSA fails to safeguard children from other common and distinct forms of abuse they may encounter in sports.

## The Current Study

Empirical evidence around sports-related CM is growing with public exposure of high-profile cases in sports. However, to date, no existing systematic review has synthesized gender-related findings of various CM subtypes in sports. To fill this knowledge gap, the primary aim of the current systematic review was to identify and synthesize peer-reviewed literature on sports-related CM to determine whether such experiences (i.e., prevalence, perpetrator characteristics, perceptions, formal and/or informal disclosure, and outcomes) differ across genders. The secondary aim was to identify implications and future directions for practice, policy, and research to better safeguard sports for children of all genders. A synthesis of this research will contribute to improvements in sports by supporting the development of gender-specific preventative and responsive CM measures (e.g., gender-informed disclosure workshops and formal reporting procedures) and informing future research to address potential gender differences in sports.

## Method

This systematic review adhered to the Preferred Reporting Items for Systematic Reviews and Meta-Analyses (PRISMA) Statement (Page et al., 2020) in the design of the review protocol and the reporting of results.

### Search Strategy

Systematic searches were conducted using three databases, PsycInfo, MedLine, and Scopus, for studies published between the earliest available date of each database up to the date of the initial search, May 7, 2024. In collaboration with a social sciences librarian, a search strategy was developed for each database: (athlete* OR sport* OR coach*) AND (“Sex* Abus*” OR “Intimate Partner Violence” OR “Sex* Harass*” OR “Sex* Violen*” OR “dating violen*” OR “Sex* Coerc*” OR rape* OR “Sex* Offen*” OR pedophil* OR “Psychosex* Behavio*r” OR “emotion* abus*” OR “verbal abus*” OR “physic* abus*” OR grooming) AND (child* OR teen* OR adolescen* OR “school age*” OR “under 18”).

### Eligibility Criteria

The following criteria were used to select studies to be included in the final analysis: (a) sample included children and adolescents (0–18 years) participating in organized sports or adults who participated in organized sports before 18, (b) included a quantitative measure of sports-related CM (e.g., CM subtype, prevalence, perpetrator characteristics, disclosure, outcomes), and (c) included a measure of and disseminated findings across genders. As the review would like to examine original data that quantified sports-related CM, studies that did not publish original data (e.g., reviews, book chapters), are not peer-reviewed (e.g., doctoral theses, conference abstracts), and where the full text is not available in English were excluded. To allow for gender comparisons, studies including only girl or only boy samples were also excluded.

### Selection Process/Screening Protocol

The PRISMA flow diagram outlines the literature search and selection process (Figure 1). The search yielded 610 articles, of which 509 unique articles remained after removing duplicates. Using prespecified eligibility criteria, three reviewers independently screened the titles and abstracts to determine relevance. Fifty-seven potentially relevant articles were identified. Subsequently, two reviewers independently screened the full text of these 57 articles against the eligibility criteria. Of those, 21 articles met all inclusion criteria. Inter-rater reliability was calculated using Cohen’s *K*, with inclusion decisions dichotomously coded as either “include” (1) or “exclude” (0). The *K* statistics for agreement between the reviewers was .85 (95% CI [0.71, 0.99], *p* < .001), indicating a strong inter-rater agreement. Additionally, a hand search of the reference lists of included studies was conducted, and no additional study was identified. However, a recently published study by Woessner et al. (2024) was recommended by a researcher in the field with whom the authors consulted. As it met all predefined inclusion criteria, it was included in the final review. Thus, the final review included a total of 22 articles.

**Figure 1.**
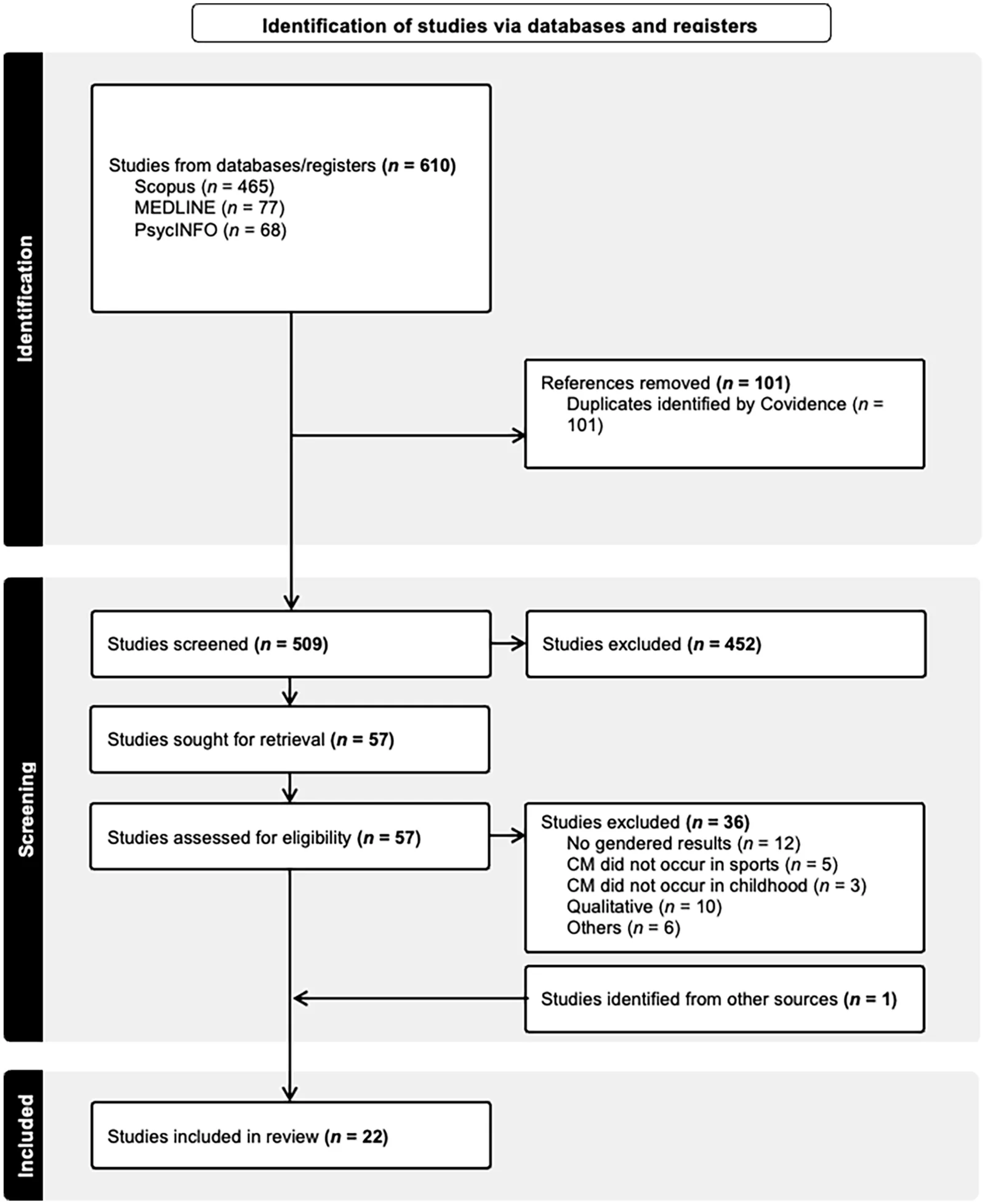
PRISMA flow diagram. *Note*. PRISMA = Preferred Reporting Items for Systematic Reviews and Meta-Analyses.

### Quality Assessment

The quality of the included studies was evaluated using the STROBE checklist, a 22-item tool (1 point for each item, for a total of 22 points; von Elm et al., 2007). The first and second author independently completed the assessment, and their results were subsequently compared. Discrepancies were resolved through discussion, with articles reread as necessary. The quality ratings of the included articles range from 15 to 21 points, indicating a moderate to high level of quality (see Supplemental Table 1). Overall, the studies included in the current review demonstrated an acceptable level of quality.

### Data Abstraction

An in-depth examination of the 22 articles meeting full eligibility criteria was conducted. A data abstraction table, specific to the aims of the review, was developed. The table included abstraction domains, including study design, sample characteristics, methodology, prevalence of sports-related CM across genders, perpetrator characteristics across genders, disclosure of sports-related CM across genders, outcomes across genders, analytic methods, key results, and study strengths and limitations. The first and second authors independently conducted data abstraction from all included articles, and the results were compared. Discrepancies were resolved through discussions between the two reviewers to reach a final consensus.

## Results

Results are synthesized and discussed according to CM subtypes. Of the 22 articles included, 18 unique samples were examined. Among these, 14 used surveys and four examined documented cases of sports-related CM. The most common questionnaire used by survey studies was the Violence Toward Athletes Questionnaire (VTAQ; used by five studies). Eight studies sampled adolescents (i.e., 12–18-year-olds), while six relied on adults’ retrospective reporting of childhood experiences. One study examined a wider age range (i.e., 4–18-year-olds; Tsuchiya et al., 2017) and one surveyed secondary school students and included a small number of participants between 19 and 21 years old who took longer to complete school (Vertommen et al., 2022). This study was included given the small number of participants over 18 and the relevant context examined (i.e., secondary school). Five studies were conducted in Canada, three in the United Kingdom (U.K.), two in Australia, and seven in other countries (i.e., Belgium, Denmark, Finland, Japan, the Netherlands, Norway, and the United States [U.S.]). Five studies collected samples from multiple countries. See Tables 1 and 2 for a summary of findings.

**Table 1.** Summary of Findings: Gender Differences in Prevalence, Perpetrator Characteristics, and Outcomes of Sports-Related CM.

Study	Sample	Methodology	Prevalence (G)	Prevalence (B)	Perpetrator (G)	Perpetrator (B)	Outcomes/Other Relevant Findings
Physical Violence							
Stafford et al., 2013 *United Kingdom*	Adults in organized sports as children (*N* = 6,124; 73% girls)	Survey (original)	23% overall 56% forced to train when injured/exhausted; 51% aggressive treatment; 13% violent treatment	26% overall 51% forced to train when injured/exhausted; 63% aggressive treatment; 24% violent treatment			
Psychological Violence/Neglect							
Boudreault et al., 2022 *Canada*	14–17-year-olds (*N* = 999; 72% girls; *M_age_* = 15.7)	Survey (VTAQ)	8%* reported weight-related psychological violence/neglect	5% reported weight-related psychological violence/neglect in sports	4–5% coach 1–3% parent	2–3% coach 1–2% parent	No gender differences for reporting at least one incident of weight-related maltreatment.
Stafford et al., 2015 *United Kingdom*	Adults in organized sport as children (*N* = 6,124; 73% girls)	Survey (original)	67% criticized about performance; 66% embarrassed/humiliated; 26% bullied; 33% criticized about weight/looks; 35% shouted/sworn at	72% criticized about performance; 64% embarrassed/humiliated; 27% bullied; 30% criticized about weight/looks; 55% shouted/sworn at	36% coach 79% peer	29% coach 87% peer	
Sexual Violence							
Brackenridge et al., 2008 *International*	Sexual abuse cases in sports between 1992 and 2006 (*N* = 159)	Case examination	31 cases with victims under 12; 84 cases with victims aged 13–17	28 cases with victims under 12; 32 cases with victims aged 13–17			CSA strategies used with girls are less aggressive
Nielsen, 2001 *Denmark*	Student athletes (*N* = 253; 33% girls)	Survey (original)	12% asked out to the movies, dinner; 34% called a pet name; 25% stared at their breasts/buttocks; 9% kissed on the mouth; 3% proposed a sexual encounter	10% asked out to the movies, dinner; 40% called a pet name; 49% stared at their breasts/buttocks; 0% kissed on the mouth; 1% proposed sexual encounter			
Parent et al., 2016 *Canada*	14–17-year-olds (weighted *n* = 6,268; 58% girls; *M_age_* = 15.4)	Survey (original)	0.4% CSA^ns^; 0.4% without penetration; 0.1% with penetration; 0.2% sexual harassment	0.7% CSA^ns^; 0.6% without penetration; 0.4% with penetration; 0.6%* sexual harassment			
Sanderson & Weathers, 2020 *International*	Media coach CSA cases involving Snapchat between 2011 and 2018 (*N* = 99; *M_age_* victims = 15)	Case examination	61% involved girls	26% involved boys	59% male coach; 2% female coach	17% male coach; 9% female coach	
Sølvberg et al., 2022 *Norway*	Elite, recreational, and non-athletes in grade 12 (*N* = 919; 51% girls; *M_age_* = 17.1)	Survey (original) examining 12-month prevalence 12 months apart.	SHA outside school Elite athletes:15% at T1, 13% at T2* Recreational athletes: 24% at T1, 22% at T2* Reference students:7% at T1, 5% at T2^ns^	SHA outside school Elite athletes: 13% at T1, 7% at T2 Recreational athletes:19% at T1, 11% at T2 Reference students: 2% at T1, 3% at T2^ns^	Most common perpetrator across groups: Male trainer/teacher, male other*	Most common perpetrator across groups: Male peers, female/both genders other*	
Timon et al., 2022 *U.S.A.*	Adults who were in elite sport as children (*N* = 473; 70% girls; *M_age_* = 29)	Survey (original)	4% of the sample (all girls) reported CSA		2% peer 2% coach		Experience of CSA sig. more likely to have a mental health diagnosis
Vertommen et al., 2015 *Netherlands*	NOC*NSF helpline incident registration forms between 2001 and 2010 (*N* = 323)	Case examination	4% involved victims under 12; 27% involved victims aged 12 -15*	5% involved victims under 12*; 17% involved victims aged 12 – 15.			Perpetrators under 16 tend to target boy victims
Multiple Forms of Violence							
Daignault et al., 2023 *Canada*	14–17-year-olds (*N* = 1,057; 72% girls; *M_age_* = 15.3)	Survey (VTAQ)	68% of non-victimization group; 76% of psychological victimization group*; 66% of the poly-victimization group* are girls	32% of the non-victimization group; 24% of the psychological victimization group; 34% of the poly-victimization group are boys			
Hartill & Lang, 2018 *United Kingdom*	Sports child protection referrals/allegations reported to local authorities between 2010 and 2015 (*N* = 1,013)	Case examination	57% involved girls; 16% victims under 10; 57% between 11 and 17-years 70% involved CSA, 14% physical, 4% emotional, 0.4% neglect, 10% others.	40% involved boys; 24% victims under 10; 50% between 11 and 17-years 35% involved CSA, 46% physical, 6% emotional, 4% neglect, 8% others.	87% male perpetrator 9% female perpetrator	83% male perpetrator 11% female perpetrator	Younger victims more likely to be boys, older victims more likely to be girls.
Hartill et al., 2023 *Austria, Belgium, Germany, United Kingdom, Spain, Romania*	Adults who were in sports as children (*N* = 10,302; 50% girls; *M_age_* = 24.4)	Survey (IVAS-Q)	71% reported one kind of sports-related CM 30% neglect; 61% psychological; 36% physical; 32% non-contact sexual; 14% contact sexual	79% reported one kind of sports-related CM* 44% neglect*; 68% psychological*; 52% physical*; 38% non-contact sexual*; 26% contact sexual*			Gender was a sig. predictor for physical violence and neglect.
Pankowiak et al., 2023 *Australia*	Adults who were in sports as children (*N* = 886; 63% girls; *M_age_* = 42)	Survey (VTAQ)	82% psychological*; 31% neglect*; 40% sexual abuse*; 40% sexual harassment*; 65% physical^ns^	74% psychological; 18% neglect; 33% sexual abuse; 33% sexual harassment; 66% physical^ns^	Psychological: 55% coach^ns^; 72% peer*; 33% parent* Neglect: 24% coach*; 17% parent* Sexual (contact): 4% coach*; 1% peer^ns^ Sexual (no contact): 5% coach Sexual harassment: 19% coach*, 32% peer^ns^ Physical: 57% coach^ns^; 23% peer;16% parent^ns^	Psychological: 50% coach^ns^; 64% peer; 23% parent Neglect: 14% coach; 7% parent Sexual (contact): 1% coach; 2% peer^ns^ Sexual (no contact): 2% coach Sexual harassment: 8% coach^ns^, 29% peer Physical: 51% coach^ns^; 39% peer*; 16% parent^ns^	Girls sig. more at risk of more than one CM subtype. Gender was not a sig. risk factor for most CM subtypes. Gender-diverse individuals are more at risk for violence from all perpetrators.
Parent & Vaillancourt-Morel, 2021 *Canada*	14–17-year-olds participating in organized sports (*N* = 1,055; 72% girls; *M_age_* = 15.3)	Survey (VTAQ)	81% psychological*; 40% neglect*; 36% physical; 29% sexual^ns^ 38% one type; 32% two types; 16% three types	75% psychological; 29% neglect; 49% physical*; 25% sexual^ns^ 33% one type; 32% two types; 19% three types			No gender difference found in number of CM subtypes experienced.
Parent et al., 2022 *Canada*	14–17-year-olds participating in organized sports (*N* = 1,055; 72% girls; *M_age_* = 15.3)	Survey (VTAQ)			68% peer; 70% coach; 44% parent; 21% one perpetrator; 30% two; 34% three	68% peer; 64% coach; 32% parent; 28% one perpetrator; 31% two; 24% three	All sports-related CM associated with mental health problems, comparable across genders. More physical abuse by peers and coaches in boys; more sexual abuse by coaches in girls.
Peltota & Kivijärvi, 2017 *Finland*	12,13,15, 16-year-olds (*N* = 11,364; 54% girls; *n* = 5,489 reported sports as their primary structured activity)	Survey (Finnish Child Victim Survey)	15% reported emotional abuse by a coach	31% reported emotional abuse by a coach.			Boys reported more experiences of overall and physical abuse from a coach.
Tsuchiya et al., 2017 *Japan*	4–18-year-old athletes (*N* = 5,735; 28% girls; *Median_age_* = 11)	Survey (original)	No dental injuries: 22% verbal abuse; 11% physical abuse With dental injuries: 27% verbal abuse; 13% physical abuse	No dental injuries: 20% verbal abuse; 12% physical abuse With dental injuries: 27% verbal abuse; 18% physical abuse			Coach verbal and physical abuse associated with more sports-related dental injuries in boys
Vertommen et al., 2022 *Belgium*	Secondary school students (*N* = 768; 33% girls; *M_age_* = 15.9)	Survey (VTAQ)	86% reported any type of violence^ns^; 1% all types^ns^ 81% psychological abuse/neglect^ns^; 41% physical; 48% instrumental^ns^; 32% sexual* 25% one type; 25% two; 21% three; 16% four	87% reported any type of violence^ns^; 2% all types^ns^ 78% psychological abuse/neglect^ns^; 60% physical*; 52% instrumental^ns^; 24% sexual 17% one type; 25% two; 29% three; 16% four	69% coach^ns^ 73% peer^ns^ 48% parent^ns^	72% coach^ns^ 69% peer^ns^ 48% parent^ns^	
Vertommen et al., 2017 *Belgium & Netherlands*	Adults who were in organized sport as children and reported sports-related CM (*N* = 1,785; 43 – 66% girls across subtypes)	Survey (IViS)			Most common for Psychological: several female athletes (20%) Physical: one male coach (24%) Sexual: one male other (21%)	Most common for Psychological: several male athletes (32%) Physical: several male athletes (21%) Sexual: several male others (20%)	Girls reported sig. more male and single perpetrator of psychological and physical abuse. Boys reported sig. more male perpetrator only abuse. Victim’s gender did not affect severity.
Vertommen et al., 2016 *Belgium & Netherlands*	Adults who were in organized sport as children (*N* = 4,043; 55% girls)	Survey (IViS)	36% psychological abuse^ns^: 10% severe^ns^ 9% physical abuse: 5% severe 17% sexual abuse*: 7% severe*	39% psychological abuse^ns^: 9% severe^ns^ 14%* physical abuse: 12% severe* 11% sexual abuse: 4% severe			

*Note*. All rates are rounded to the nearest whole number. G = girl; B = boys; CSA = child sexual abuse; SHA = sexual harassment and abuse; sig. = significantly; T = time.

* Statistically significantly greater compared to other gender; ^ns^non-statistically significant gender difference.

**Table 2.** Summary of Findings: Gender Differences in Disclosure and Perception of Sports-Related CM.

Study	Sample	Methodology	Findings: Girls	Findings: Boys
Parent et al., 2016 *Canada*	14–17-year-olds (weighted *n* = 6,268; 58% girls)	Survey (original)	32% of overall sample, 41% of athlete sample, perceived sexual contacts with a coach as consensual.	68%* of overall sample, 69% of athlete sample, perceived sexual contacts with a coach as consensual.
Timon et al., 2022 *U.S.A.*	Adults who were in elite organized sport as children (*N* = 473; 70% girls; *M_age_* = 29)	Survey (original)	No participants disclosed.	
Woessner et al., 2023 *Australia*	Adults who were in community sport as children (*N* = 886; *M_age_* = 42)	Survey (VTAQ)	38% disclosed peer-perpetrated CM*. Top recipients: parent (93%), coach (39%) 29% disclosed coach-perpetrated CM^ns^. Top recipients: parent (96%), sport club manager (12%) 13% disclosed parent-perpetrated CM^ns^. Top recipients: parent (63%), adult sibling (17%)	28% disclosed peer-perpetrated CM. Top recipients: parent (77%), coach (58%) 23% disclosed coach-perpetrated CM^ns^. Top recipients: parent (84%), coach/sport club manager (19%) 13% disclosed parent-perpetrated CM^ns^. Top recipients: parent (80%), coach/sport club manager (50%)

*Note*. All rates are rounded to the nearest whole number. CM = child maltreatment.

* Statistically significantly greater compared to other gender; ^ns^non-statistically significant gender difference.

### Terminologies

The current review utilizes the term “gender” to denote the social construct of how individuals perceive themselves. Provided the nature of self-report studies, gender may or may not be congruent with participants’ biological sex. However, while the current review utilizes this guiding definition and reports gendered findings as described by the included studies, not all studies measured or defined gender the same way. While several studies included gender-diverse respondents (e.g., non-binary, questioning) only one recruited a sufficient number to report statistical findings (Pankowiak et al., 2023).

Definitions of sports-related CM varied throughout the included articles. First, 13 studies considered and measured peer-perpetrated violence. Parent-perpetrated violence was only measured by studies using the VTAQ (i.e., five studies). All studies examined coach-perpetrated violence. Next, two areas of important disagreement were observed in the operationalization of physical and sexual abuse. Some researchers adopted a definition of physical abuse that considered both acts of actual (i.e., physical contact, such as hitting or pushing) and implied/potential physical violence (i.e., no physical contact but leads to physical harm, such as overtraining) (e.g., Stafford et al., 2013; Vertommen et al., 2017). All studies that adopted the VTAQ, on the other hand, categorized acts without physical contact that may lead to physical harm as *psychological* violence. A similar discrepancy was noted in sexual abuse. Most authors measured both contact (e.g., sexual intercourse) and non-contact (e.g., voyeurism) sexual abuse. Exceptionally, Timon et al. (2022) excluded the measure of non-contact sexual abuse, as acts such as exposure to genitals may be a common occurrence in locker rooms. Although the current review reported findings with the terms originally used by the authors, this discrepancy in what constitutes each CM subtype must be considered when interpreting the results.

As not all studies included statistical comparisons across genders, statistically significant gender findings in prevalence rates are reported in Table 3.

**Table 3.** Significant Gender Differences in Prevalence by CM Subtype (No. of Studies).

CM Subtype	Significantly More Prevalent for Girls	Significantly More Prevalent for Boys	Non-Significant Gender Differences
Physical Abuse	0	4	1
Psychological Abuse	4	1	2
Neglect	3	1	1
Sexual Abuse	4	1	2
Overall/Any subtype	1	1	2

### Physical Abuse

One study examined gender differences in physical abuse (Stafford et al., 2013). Specifically, the authors measured coach-perpetrated overtraining/training through injury, physical aggression, and violent treatment. Here, boys reported more overall coach-perpetrated physical abuse and more aggressive (e.g., shoved, shaken) and violent (e.g., hit with an open hand or fist) treatments, while girls reported more experiences of being forced to train when exhausted or injured.

### Psychological Abuse/Neglect

Psychological abuse and neglect were compared across genders in two studies (Boudreault et al., 2022; Stafford et al., 2015). Stafford et al. (2015) found that more boys reported being criticized about their performance and being shouted/sworn at. On the other hand, Boudreault et al. (2022) found that girls reported significantly more weight-related psychological maltreatment or neglect (i.e., being forced to or adults knowingly not intervening in the use of extreme methods to reach the ideal weight for their sport) from coaches or parents, although both genders were similarly likely to report at least one instance of such abuse. Regarding perpetrators, girls reported more coach-perpetrated psychological abuse than boys, while boys reported more peer-perpetrated psychological abuse than girls (Stafford et al., 2015).

### Sexual Abuse

Sexual abuse was the most common CM subtype to be examined, with seven studies comparing it across genders. However, how sexual abuse was measured and defined varied. Three studies (Nielsen, 2001; Sølvberg et al., 2022; Vertommen et al., 2015) examined SHA (i.e., a broader definition which measures a continuum of sexual harassment, such as repeated, unwanted sexual remarks, to abusive, like proposed sexual encounter or rape, experiences). Three studies (Brackenridge et al., 2008; Sanderson & Weathers, 2020; Timon et al., 2022) examined only the most severe form, CSA. Parent et al. (2016) was the only article that distinguished and measured SHA separately.

Three studies examined cases or allegations of CSA and SHA in various settings, and all identified more cases involving girl victims (Brackenridge et al., 2008; Sanderson & Weathers, 2020; Vertommen et al., 2015). Even so, gender differences emerged. SHA victims under 12 were significantly more likely to be boys, while older victims were significantly more likely to be girls (Vertommen et al., 2015). CSA strategies used with girls were found to be less aggressive and more intimate (e.g., declaring love, kissing) while strategies used with boys were more aggressive and intrusive (e.g., providing illicit goods or pornography, buggery/anal intercourse) (Brackenridge et al., 2008). Regarding perpetrators, boys were more likely to be victims of perpetrators under 16 years of age (Vertommen et al., 2015).

The remaining studies surveyed adolescents and adults. Measuring both peer- and coach-perpetrated SHA, girls reported a significantly higher 12-month prevalence than boys (Sølvberg et al., 2022). A different pattern was observed when perpetrators were limited to coaches, where boys were significantly more likely to report experiences of sexual harassment (Parent et al., 2016). SHA strategies used by coaches varied, with boy athletes reporting more experiences of their coach making sexist jokes or staring at their breasts/buttocks, while girl athletes reported more frequent experiences of being kissed on the mouth (Nielsen, 2001). Only one study examined various SHA perpetrators and found that coaches are the most common perpetrators for girls, and boy peers are the most common perpetrators for boys (Sølvberg et al., 2022).

In comparison, CSA prevalence was lower across genders. Coach-perpetrated CSA did not differ across genders (Parent et al., 2016). However, when including peer perpetrators, all victims in Timon et al.’s (2022) survey were girls, with approximately equal number of respondents reporting a peer or a coach as perpetrators.

Outcomes were reported by one study, indicating an increased risk of mental health diagnoses (Timon et al., 2022). Gender differences were not reported as all victims were girls.

### Multiple Forms of Maltreatment

Eleven studies measured multiple forms of sports-related CM (i.e., various CM subtypes were measured within the sample). The VTAQ was used to measure all sports-related CM subtypes (i.e., physical, psychological/neglect, and sexual) in five studies, and other surveys were used in four studies. Apart from one (Peltola & Kivijärvi, 2017), all examined sports-related CM perpetrated by coaches, parents, and peers. One study measured physical and psychological CM (Tsuchiya et al., 2017), and one reviewed child protection allegations (Hartill & Lang, 2018).

#### Physical Abuse

Four articles found boys to be significantly more at risk for physical abuse (Hartill et al., 2023; Parent & Vaillancourt-Morel, 2021; Vertommen et al., 2016, 2022). Examination of child protection allegations also found that more boy cases involve physical abuse (Hartill & Lang, 2018). No significant gender difference was identified in one article (Pankowiak et al., 2023).

The most common perpetrator of physical abuse in sports was a coach (Pankowiak et al., 2023), specifically a male coach (Vertommen et al., 2017), for girls, and a coach (Pankowiak et al., 2023) or same-sex peers (Vertommen et al., 2017) for boys. Compared to girls, boys were found to be more at risk for physical abuse at the hands of their peers (Pankowiak et al., 2023; Parent et al., 2022) and coaches (Parent et al., 2022). Gender-diverse individuals reported significantly higher rates of physical abuse from coaches, peers, and parents compared to gender-conforming children (Pankowiak et al., 2023).

#### Psychological Abuse/Neglect

Girls (Daignault et al., 2023; Pankowiak et al., 2023; Parent & Vaillancourt-Morel, 2021) and gender-diverse children (Pankowiak et al., 2023) were significantly more at risk for psychological abuse. Two studies did not find gender differences (Vertommen et al., 2016, 2022), and one found that gender was not a significant predictor, despite boys reporting significantly higher rates of this abuse (Hartill et al., 2023). Neglect was less consistent, where boys were significantly more at risk in one study (Hartill et al., 2023), but Australian (Pankowiak et al., 2023) and Canadian (Parent & Vaillancourt-Morel, 2021) girls were significantly more at risk in two. No gender differences were identified in one study (Vertommen et al., 2022).

Peers (Pankowiak et al., 2023), especially same-sex peers (Vertommen et al., 2017), were identified as the most common perpetrators of psychological abuse in sports for both genders, while coaches were the main perpetrators of sports-related neglect for both genders (Pankowiak et al., 2023). Girls, however, reported significantly more psychological abuse from peers, parents, and coaches, and more neglect from coaches and parents compared to boys (Pankowiak et al., 2023; Parent et al., 2022). Gender-diverse individuals were significantly more at risk of psychological abuse from coaches compared to boys and girls (Pankowiak et al., 2023).

#### Sexual Abuse

Girls were significantly more at risk for sexual abuse in three studies (Pankowiak et al., 2023; Vertommen et al., 2016, 2022) and for sexual harassment in one study (Pankowiak et al., 2023). Similarly, victims of CSA cases were two times more likely to be girls (Hartill & Lang, 2018). No gender difference was found in one study (Parent & Vaillancourt-Morel, 2021), and gender was not a significant predictor in Hartill et al. (2023) albeit higher rates reported among boys.

Peers (Pankowiak et al., 2023) or male staff (e.g., medical staff, board members, referees, and other sports personnel; Vertommen et al., 2017) were the most common perpetrators of sports-related sexual abuse against both genders. However, girls were significantly more at risk for coach-perpetrated sexual abuse and harassment (Pankowiak et al., 2023). Compared to gender-conforming children, gender-diverse individuals reported significantly higher rates of sexual abuse perpetrated by coaches and peers (Pankowiak et al., 2023).

#### Outcomes and Other Findings

Boys reported significantly more sports-related CM overall (any type) in one study (Hartill et al., 2023), but both genders were at similar risk in two studies (Pankowiak et al., 2023; Vertommen et al., 2022). In Hartill and Lang’s (2018) review of child protection referrals, younger victims (i.e., under 10 years old) were more likely to be boys, whereas older victims (i.e., 11–17 years old) were more likely to be girls. The number of CM subtypes experienced did not differ across genders in one study (Parent & Vaillancourt-Morel, 2021), while Pankowiak et al. (2023) found girls to be significantly more at risk for experiencing more than one type of sports-related CM. Further, across CM subtypes, girls and boys reported comparable rates of peer-, coach-, and parent-perpetrated CM in sports (Vertommen et al., 2022).

Consequences of various sports-related CM were measured and compared across genders in two studies (Parent et al., 2022; Tsuchiya et al., 2017). All subtypes of sports-related CM were associated with serious mental health consequences (i.e., lower self-esteem, higher psychological distress, and PTSD symptoms), and these associations were largely comparable across genders with few exceptions (Parent et al., 2022). Specifically, the links between coach-perpetrated psychological abuse/neglect and self-esteem or distress, as well as between abuse from parents or multiple perpetrators and PTSD symptoms, were significantly stronger for girls (Parent et al., 2022). Further, verbal and physical abuse from coaches was positively associated with a higher prevalence of sports-related dental injuries in boys, but not girls, in Japan (Tsuchiya et al., 2017).

### Perceptions and Disclosures of Sports-Related Child Maltreatment

Perception of sports-related CM, specifically sexual abuse, was examined by one study (Parent et al., 2016). Among adolescents who reported sexual contact with a coach, boys were significantly more likely to perceive such contact as consensual (Parent et al., 2016).

Two studies measured the disclosures of sports-related CM. In Timon et al.’s (2022) survey, only two participants (both girls) responded to disclosure questions, and neither disclosed CSA experienced in sports. One noted that she “did not know where to go or what to do” (Timon et al., 2022). Finally, Woessner et al. (2024) examined disclosure rates of sports-related CM reported in Pankowiak et al. (2023). Significantly fewer boys disclosed peer-perpetrated violence, and no gender differences were found for the disclosure rates of coach- and parent-perpetrated violence. Importantly, who the children told differed. Girls almost always disclosed peer-perpetrated violence to a parent, whereas boys were significantly more likely to turn to a coach. For parent-perpetrated violence, girls were significantly less likely to disclose to their coaches, preferring to tell another family member, such as another parent or a sibling, whereas boys told their parents and coaches. No gender differences were found for coach-perpetrated violence. Both boys and girls primarily told their parents or sports club managers, although boys also frequently disclosed to their coaches.

## Discussion

This systematic review was the first to synthesize existing research to examine gender differences in various forms of sports-related CM. Twenty-two articles compared experiences or disclosures of physical, psychological, and sexual abuse and neglect in sports across genders. Several gender-specific insights and implications for research and policies aimed at safeguarding sports for children were identified.

### Terminologies and Demographics

The current review found important discrepancies in how subtypes of CM are operationalized in sports (i.e., contact/non-contact physical and sexual abuse). A lack of consensus on the definitions and measurement tools makes it difficult to compare sports-related CM experiences across countries, sports, and genders, or to protect children through reliable and legal identification, investigation, and prosecution of such maltreatment. These inconsistencies must be considered when interpreting the findings of the current review. That is, the observed gender differences, or the absence of them, may partly reflect variation in how included studies defined, measured, and reported experiences of sports-related CM. Efforts to propose concrete manifestations of each CM subtype in sports, such as Fortier et al. (2020) and the International Olympic Committee consensus (Tuakli-Wosnornu et al., 2024), are important first steps toward consistent measurement for both research and legal practice. It is imperative that researchers carefully examine these efforts when conducting future studies on related topics and that practitioners and policy-makers consider the same definitions for consistency.

Limitations in participant demographics were further identified. Most articles in the current review surveyed adolescents (i.e., 12–18 years old) or relied on retrospective recall from adults. However, findings indicated that boys make up most of the youngest victims (i.e., under 12) referred to helplines (Vertommen et al., 2015) and child protection agencies (Hartill & Lang, 2018). By excluding children under 12, current self-report studies may underestimate the prevalence and risks the youngest boy athletes face and fail to identify whether the dynamics of abuse differ across age groups. Future self-report studies should examine a wider age range, especially for boys. Further, all but one study in the current review is conducted in the global West. Although Canadian and European studies are well represented, sports-related CM has scantly been examined in the United States where numerous high-profile cases, such as the Larry Nassar case, originated (e.g., Mountjoy, 2019). It is concerning that this media attention has not been matched with empirical attention. Limited diversity of participants is another concern. While several studies in the current review included quantitative data on gender-diverse individuals, only one reported quantitative data for this group (Pankowiak et al., 2023). Future research should not only strive to include gender-diverse and transgender children, but also report descriptive findings wherever possible, even if statistical comparisons with other gender groups are not feasible due to limited sample size. No studies in the review explored intersectionality (i.e., how gender interacts with other risk factors such as disability and race/ethnicity). While some studies looked at these risk factors independently (e.g., Parent & Vaillancourt-Morel, 2021; Vertommen et al., 2016), number of participants belonging to minority groups was low (e.g., 8.3% of Vertommen et al.’s [2016] study identified as an ethnic minority, 4.6% participated in sports for disabled children, 92.5% of Parent and Vaillancourt-Morel’s [2021] study identified as heterosexual). Whether minority boys and girls experience CM in sports differently must continue to be explored in diverse samples.

### Gender Differences in Prevalence, Perpetrator, Outcomes, and Perceptions/Disclosures

The findings highlight several gender differences concerning the prevalence, perpetrator characteristics, outcomes, and perceptions/disclosures of sports-related CM. First, consistent with findings outside of sports (Moody et al., 2018), girls reported a greater prevalence of neglect and psychological and sexual abuse in most studies. On the other hand, boys reported higher rates of physical abuse in sports, although rates were comparable across genders outside of sports (Moody et al., 2018). This discrepancy is likely due to the current review’s inclusion of peer-perpetrated maltreatment. It was found that although coaches were the most common perpetrators of physical abuse against girls, both peers and coaches emerged as common perpetrators against boys. In fact, Vertommen et al. (2017) found that physical abuse against boys was most often perpetrated by *several* boy athletes. Such findings reflect the proposal that the hypermasculinization of sports encourages the use of physical domination and aggression as a means to perform masculinity (see Kreager, 2007). Men athletes and coaches may have normalized using physical aggression as a way to intimidate, maintain, and demonstrate their masculine identities. Further investigation regarding how to disrupt such performance of masculinity in men sports is needed, such as the role of positive men role models.

The current findings also highlight concerns about underreporting among both genders. Psychological abuse was the most prevalent CM subtype for both boys and girls in all studies examining multiple forms of maltreatment, but it only makes up a minority of child protection referrals (e.g., Hartill & Lang, 2018). This is especially alarming for girls, who are at a much higher risk of this form of abuse. Qualitative research indicated that psychologically abusive behaviors are normalized in women sports as a way to develop toughness or enhance performance (Wilinsky & McCabe, 2021). Although they lead to detrimental consequences (e.g., impacts on self-esteem and mental health; Wilinsky & McCabe, 2021), athletes, especially female athletes, may consider them a part of the game and do not find them worthy of reporting to officials. Its prevalence across genders and the lack of the boys’ perspective call for more quantitative research examining gender-specific barriers to reporting this form of abuse. The finding that boys reported a lower rate of sexual abuse in sports also reflects clinical and empirical evidence regarding the consistent underreporting of CSA among boy victims both in (Parent & Bannon, 2012) and out (e.g., Lev-Wiesel & First, 2018) of sports. The observations that boys were more likely to perceive sexual contact with their adult coaches as consensual (Parent et al., 2016) and reported more aggressive CSA strategies in sports (Brackenridge et al., 2008) further correspond with some of the most common barriers boys face in disclosing CSA outside of sports (i.e., downplaying or not recognizing their experience as abusive; Alaggia et al., 2019). Like the broader boy population, boy athletes may be more likely to perceive intimate experiences as consensual, while only perceiving an act as abusive when the perpetrators are aggressive. However, the current review did not identify gendered findings regarding how boys and girls might differ in their perception or disclosure of CSA in sports. This forms another important area for future examination.

Differences in the common perpetrators and disclosure recipients of sports-related CM were also observed. Overall, boys reported more peer-perpetrated, whereas girls reported more coach-perpetrated, sports-related CM of all forms (Woessner et al., 2024). This may explain why compared to boys, girls are less likely to disclose sports-related CM to their coaches, preferring to turn to their parents instead (Woessner et al., 2024). Yet, the few studies that examined child protection allegations and referrals identified fewer cases involving boy victims (e.g., Hartill & Lang, 2018). The parental role of coaches and the value boy athletes place in their sports is evident through these findings. However, coaches continue to be common perpetrators of sports-related CM against boys (e.g., Vertommen et al., 2022) and potential contributors to dead-end disclosures. Could it be that, unlike parents, coaches more often fail to report the disclosures they receive from boy athletes? Sports institutions were found to have few policies and procedures to support CM disclosures, and stakeholders, including coaches, were either unaware or unequipped to deal with these complaints (Parent, 2011). Coaches who receive CM disclosures also face the dilemma of having to choose between believing the athlete or their colleague (or other athletes on their team) (Parent, 2011). Under this pressure, they may prefer to stay silent and let things blow over (Parent, 2011). Such inaction contributes to the normalization of CM in sports, allows perpetrators to remain in sports, and hinders further disclosures from victims.

Finally, the results of the included studies point to one area of congruency. Although boys and girls reported different prevalences and perpetrators of sports-related CM, the consequences of experienced maltreatment are comparable across genders. In other words, regardless of the gender differences found, young athletes are reporting serious and consequential CM experiences in sports. Concerningly, only three of the included studies compared outcomes and severity of CM across genders, which underscores the need for more research in this area. For instance, included studies only examined the internalizing outcomes of sports-related CM (e.g., mental health). Outside sports, it has been found that maltreated boys are more likely to develop externalizing behaviors and be involved with the justice system due to violent offences (Baidawi et al., 2023). The high rate of peer-perpetrated violence against boys found in the current review and previous findings indicating a strong relationship between sports involvement and violence among boys (Kreager, 2007) point to a potential victim-perpetrator profile among boy athletes. In other words, are boys who were victims of sports-related CM more likely to also perpetrate violence against their peers? Future research examining gender-specific externalizing behaviors as outcomes of sports-related CM is needed. This understanding will be crucial for the development and implementation of targeted support for boy victims of CM in sports and, in turn, protect boy athletes from peer-perpetrated violence. Regardless, findings indicate that children of all genders are reporting serious outcomes of CM encountered in sports, highlighting the dire need for both empirical and applied efforts to safeguard sports for young athletes.

### Implications

A summary of the implications for practice, policy, and research drawn from the current findings can be found in Table 4. First, the findings regarding gender-specific underreporting of sports-related CM should be of interest to policy-makers and practitioners, as they point to the need for *gender-specific measures*. Although child protection initiatives in sports have placed a heavy emphasis on awareness raising through educational resources (Kerr et al., 2014), these resources will benefit from catering to gendered needs. Resources for boys should be more explicit regarding the age of consent and grooming (e.g., intimate acts can also be abusive), whereas resources for girls should deconstruct the norm of using threats and humiliation to enhance athletic performance. Gender-based strategies, supported by empirical research, may be more effective in facilitating CM identification and disclosure in sports.

**Table 4.** Implications for Policy, Practice, and Research.

Implications	
Research	Establish consistent definitions and measurement of CM subtypes in sports. Examine a wider age range and diversity of participants Examine how different genders perceive each CM subtype and their unique barriers to disclosing each CM subtype in sports
Practice	Conduct regular audits evaluations regarding compliance and knowledge of sports-related CM identification and reporting in sport institutions
Policy	Develop formal reporting procedures within sports institutions and ensure all stakeholders are aware of their duty to report and to understand the reporting structure Mandatory trainings for coaches regarding how to support, guide, and make formal reports if CM disclosures are received, especially for coaches of boys’ sports Develop and employ gender-specific educational resources for identifying and reporting sports-related CM (e.g., emphasis on consent and grooming for boys and psychological violence and overtraining for girls).

Another implication relates to gender differences in the disclosure of sports-related CM. The development of formal reporting procedures in sports and mandatory training for coaches regarding appropriate next steps when they receive disclosures of maltreatment is critical. These trainings must recognize the role coaches play in athletes, especially boy athletes’ lives, increase coach awareness in their role as recipients of sports-related CM disclosures, and equip them with the necessary skills and knowledge to support their athletes (e.g., how to protect athletes’ identity and decision to remain anonymous, where to report). Empirical research must further support the development and evaluation of these reporting procedures and training. That is, systemic and regular evaluations of whether athletes, coaches, and others involved in sports are truly aware and compliant with such procedures are necessary to ensure real change.

## Limitations and Strengths

Several limitations must be considered when interpreting the results of the current review. First, only studies published in English were included, which may have excluded significant research conducted in other languages. In line with this, while the search strategy was designed to capture a wide range of relevant studies, terminological and database choices may have excluded studies that examined sports-related CM under different terminology. Second, the majority of studies included relied on self-reporting from adolescents or retrospective recall from adults. As noted, adolescents face a variety of challenges that prevent disclosure, even when anonymous. These barriers further differ across genders. It is therefore likely that most data underreport the true rates of sports-related CM. Similarly, there is an absence of perspectives from children under 12. While it is important not to exclude younger children from this research, there exists significant ethical and legal challenges in asking them about their experiences of maltreatment in sports (e.g., mandatory reporting obligations, distress). These complexities must be navigated carefully in future research with this population. A third, and related limitation, is that not all studies included a representative sample and vary in sample sizes. For example, there is an uneven ratio of boys and girls in most studies, with many including a higher proportion of women respondents. Thus, gender differences may not be generalizable to the real, men-dominated sports context. The restriction of gender to a binary concept in almost all available research and within sports further means that a key research gap exists concerning the experiences and perceptions of gender-diverse children who participate in sports. Moreover, gender was not defined and measured consistently across included studies, with some studies not distinguishing between gender and biological sex. Thus, current findings could reflect a mixture of both sex and gender differences. Lastly, the current study is not able to differentiate whether the gender differences observed may be attributed to other underlying factors (e.g., specific sports culture). Future research should examine gender differences across factors, such as team/individual or contact/non-contact sports, to better understand the true nature of the differences observed here.

The current review also has several strengths, including adherence to a recommended systematic review protocol and systematic search and screening procedures. Importantly, this review synthesized and interpreted included studies in a gender-informed manner. While previous reviews have been conducted on the experiences (Bjørnseth & Szabo, 2018) and enabling factors (Dodd et al., 2024) of sports-related CSA and general knowledge of CM in sports (Parent & Fortier, 2017, 2018), the results in these previous reviews were limited to a specific CM subtype or the combined experiences of boy and girl athletes. The current review adds to the body of existing literature by examining the unique experiences and disclosure of CM in sports for each gender, including gender-diverse athletes where available. It is important to highlight how boys and girls differ in sports and utilize that knowledge to prevent and intervene in all CM in sports.

## Conclusion

Drawing together empirical literature on the prevalence, perpetrators, outcomes, and disclosures of sports-related CM, this systematic review aimed to examine whether these experiences differ across genders. The findings highlight gender differences in the type of CM children experienced, who the perpetrators were, and who the children told. Despite these differences, sports-related CMs bring about serious consequences and are likely underreported by both boys and girls. These findings should act as a call for research to examine gender differences in how children perceive and make the decision to disclose sports-related CM, as well as support the development of gender-informed policies and practices to prevent, identify, and facilitate disclosures of maltreatment in this unique context. This knowledge will be crucial to safeguard and ensure children of all genders continue to benefit from their sports participation.

## Supplemental Material

sj-docx-1-tva-10.1177_15248380251372157 – Supplemental material for Gender Differences in Sports-Related Child Maltreatment: A Systematic Review Supplemental material, sj-docx-1-tva-10.1177_15248380251372157 for Gender Differences in Sports-Related Child Maltreatment: A Systematic Review by Annie Yun An Shiau, Kayla D. Schick, Kelly McWilliams and Shanna Dewit-Williams in Trauma, Violence, & Abuse

## Notes

1. For the definition of gender examined in the current review, please see the “Results” section

## References

[bibr1-15248380251372157] Alaggia R. Collin-Vézina D. Lateef R. (2019). Facilitators and barriers to child sexual abuse (CSA) disclosures: A research update (2000–2016). Trauma, Violence, & Abuse, 20(2), 260–283. 10.1177/1524838017697312 PMC6429637 29333973

[bibr2-15248380251372157] Baidawi S. Papalia N. Featherston R. (2023). Gender differences in the maltreatment-youth offending relationship: A scoping review. Trauma, Violence, & Abuse, 24(2), 1140–1156. 10.1177/15248380211052106 PMC10012399 34907817

[bibr3-15248380251372157] Bjørnseth I. Szabo A. (2018). Sexual violence against children in sports and exercise: A systematic literature review. Journal of Child Sexual Abuse, 27(4), 365–385. 10.1080/10538712.2018.1477222 29877758

[bibr4-15248380251372157] Black L. I. Terlizzi E. P. Vahratian A. (2022). Organized sports participation among children aged 6–17 years: United States, 2020. NCHS Data Brief, (441), 1–8. 10.15620/cdc:119026 35969661

[bibr5-15248380251372157] Boudreault V. Gagnon-Girouard M.-P. Carbonneau N. Labossière S. Bégin C. Parent S. (2022). Extreme weight control behaviors among adolescent athletes: Links with weight-related maltreatment from parents and coaches and sport ethic norms. International Review for the Sociology of Sport, 57(3), 421–439. 10.1177/10126902211018672 PMC8928425 35310918

[bibr6-15248380251372157] Brackenridge C. H. (1994). Fair play or fair game? Child sexual abuse in sport organisations. International Review for the Sociology of Sport, 29(3), 287–298. 10.1177/101269029402900304

[bibr7-15248380251372157] Brackenridge C. H. Bishopp D. Moussalli S. Tapp J. (2008). The characteristics of sexual abuse in sport: A multidimensional scaling analysis of events described in media reports. International Journal of Sport and Exercise Psychology, 6(4), 385–406. 10.1080/1612197X.2008.9671881

[bibr8-15248380251372157] Daignault I. Deslauriers-Varin N. Parent S. (2023). Profiles of teenage athletes’ exposure to violence in sport: An analysis of their sport practice, athletic behaviors, and mental health. Journal of Interpersonal Violence, 38(11–12), 7754–7779. 10.1177/08862605221148216 PMC10170556 36748675

[bibr9-15248380251372157] Demers E. Lessard G. Parent S. Paradis H. (2021). Athlete victims of sexual violence: links to conformity to the sport ethic. European Journal for Sport and Society, 20(2), 101–117. https://doi.org/10.1080/16138171.2021.2003056

[bibr10-15248380251372157] Dodd K. Solomon C. Naughton M. Salmon P. M. McLean S. (2024). What enables child sexual abuse in sport? A systematic review. Trauma, Violence, & Abuse, 25(2), 1599–1613. 10.1177/15248380231190666 37614195

[bibr11-15248380251372157] Eime R. M. Young J. A. Harvey J. T. Charity M. J. Payne W. R. (2013). A systematic review of the psychological and social benefits of participation in sport for children and adolescents: Informing development of a conceptual model of health through sport. International Journal of Behavioral Nutrition and Physical Activity, 10(1), 98. 10.1186/1479-5868-10-98 PMC3751802 23945179

[bibr12-15248380251372157] Fortier K. Parent S. Lessard G. (2020). Child maltreatment in sport: Smashing the wall of silence: A narrative review of physical, sexual, psychological abuses and neglect. British Journal of Sports Medicine, 54(1), 4–7. 10.1136/bjsports-2018-100224 31653778

[bibr13-15248380251372157] Gallo E. A. G. Munhoz T. N. Loret de Mola C. Murray J. (2018). Gender differences in the effects of childhood maltreatment on adult depression and anxiety: A systematic review and meta-analysis. Child Abuse & Neglect, 79, 107–114. 10.1016/j.chiabu.2018.01.003 29428878

[bibr14-15248380251372157] Harrison P. (2022, May 12). “Secrets kill”: The harrowing drama about sexual abuse in football. The Guardian. https://www.theguardian.com/tv-and-radio/2022/may/12/floodlights-secrets-kill-the-harrowing-drama-about-sexual-abuse-in-football-barry-bennell

[bibr15-15248380251372157] Hartill M. Lang M. (2018). Reports of child protection and safeguarding concerns in sport and leisure settings: An analysis of English local authority data between 2010 and 2015. Leisure Studies, 37(5), 479–499. 10.1080/02614367.2018.1497076

[bibr16-15248380251372157] Hartill M. Rulofs B. Allroggen M. Demarbaix S. Diketmüller R. Lang M. Martin M. Nanu I. Sage D. Stativa E. Kampen J. Vertommen T. (2023). Prevalence of interpersonal violence against children in sport in six European countries. Child Abuse & Neglect, 146, 106513. 10.1016/j.chiabu.2023.106513 37931542

[bibr17-15248380251372157] Kerr G. Kidd B. Donnelly P. (2020). One step forward, two steps back: The struggle for child protection in Canadian sport. Social Sciences, 9(5), Article 5. 10.3390/socsci9050068

[bibr18-15248380251372157] Kerr G. Stirling A. E. Heron A. L. MacPherson E. A. Banwell J. M. (2015). The importance of touch in sport: Athletes’ and coaches’ reflections. International Journal of Social Science Studies, 3(4), 56–68.

[bibr19-15248380251372157] Kerr G. Stirling A. MacPherson E. (2014). A critical examination of child protection initiatives in sport contexts. Social Sciences, 3(4), Article 4. 10.3390/socsci3040742

[bibr20-15248380251372157] Kirby S. L. Greaves L. Hankivsky O. (2002). Women under the dome of silence: Sexual harassment and abuse of female athletes. Canadian Woman Studies, 21(3), 132–139.

[bibr21-15248380251372157] Kreager D. A. (2007). Unnecessary roughness? School sports, peer networks, and male adolescent violence. American Sociological Review, 72(5), 705–724.

[bibr22-15248380251372157] Lev-Wiesel R. First M. (2018). Willingness to disclose child maltreatment: CSA vs other forms of child abuse in relation to gender. Child Abuse & Neglect, 79, 183–191. 10.1016/j.chiabu.2018.02.010 29477611

[bibr23-15248380251372157] McPherson L. Atkins P. Cameron N. Long M. Nicholson M. Morris M. E. (2016). Children’s experience of sport: What do we really know? Australian Social Work, 69(3), 348–359. 10.1080/0312407X.2015.1055508

[bibr24-15248380251372157] Moody G. Cannings-John R. Hood K. Kemp A. Robling M. (2018). Establishing the international prevalence of self-reported child maltreatment: A systematic review by maltreatment type and gender. BMC Public Health, 18(1), 1164. 10.1186/s12889-018-6044-y PMC6180456 30305071

[bibr25-15248380251372157] Mountjoy M. (2019). “Only by speaking out can we create lasting change”: What can we learn from the Dr Larry Nassar tragedy? British Journal of Sports Medicine, 53(1), 57–60. 10.1136/bjsports-2018-099403 29936429

[bibr26-15248380251372157] Nielsen J. T. (2001). The forbidden zone: Intimacy, sexual relations and misconduct in the relationship between coaches and athletes. International Review for the Sociology of Sport, 36(2), 165–182. 10.1177/101269001036002003

[bibr27-15248380251372157] Page M. J. McKenzie J. Bossuyt P. Boutron I. Hoffmann T. Mulrow C. Shamseer L. Tetzlaff J. Akl E. Brennan S. E. Chou R. Glanville J. Grimshaw J. Hróbjartsson A. Lalu M. M. Li T. Loder E. Mayo-Wilson E. McDonald S. . . . Moher D. (2020). The PRISMA 2020 statement: An updated guideline for reporting systematic reviews. http://dx.doi.org/10.1016/j.jclinepi.2021.03.001 10.1016/j.jclinepi.2021.03.001 33789819

[bibr28-15248380251372157] Pankowiak A. Woessner M. N. Parent S. Vertommen T. Eime R. Spaaij R. Harvey J. Parker A. G. (2023). Psychological, physical, and sexual violence against children in Australian community sport: Frequency, perpetrator, and victim characteristics. Journal of Interpersonal Violence, 38(3–4), 4338–4365. 10.1177/08862605221114155 35944902

[bibr29-15248380251372157] Parent S. (2011). Disclosure of sexual abuse in sport organizations: A case study. Journal of Child Sexual Abuse, 20(3), 322–337. 10.1080/10538712.2011.573459 21660817

[bibr30-15248380251372157] Parent S. Bannon J. (2012). Sexual abuse in sport: What about boys? Children and Youth Services Review, 34(2), 354–359. 10.1016/j.childyouth.2011.11.004

[bibr31-15248380251372157] Parent S. Demers G. (2011). Sexual abuse in sport: A model to prevent and protect athletes. Child Abuse Review, 20(2), 120–133. 10.1002/car.1135

[bibr32-15248380251372157] Parent S. Fortier K. (2017). Prevalence of interpersonal violence against athletes in the sport context. Current Opinion in Psychology, 16, 165–169. 10.1016/j.copsyc.2017.05.012 28813343

[bibr33-15248380251372157] Parent S. Fortier K. (2018). Comprehensive overview of the problem of violence against athletes in sport. Journal of Sport and Social Issues, 42(4), 227–246. 10.1177/0193723518759448

[bibr34-15248380251372157] Parent S. Lavoie F. Thibodeau M.-È. Hébert M. Blais M. & Team PAJ. (2016). Sexual violence experienced in the sport context by a representative sample of Quebec adolescents. Journal of Interpersonal Violence, 31(16), 2666–2686. 10.1177/0886260515580366 PMC5067151 25873593

[bibr35-15248380251372157] Parent S. Vaillancourt-Morel M.-P. (2021). Magnitude and risk factors for interpersonal violence experienced by Canadian teenagers in the sport context. Journal of Sport and Social Issues, 45(6), 528–544. 10.1177/0193723520973571

[bibr36-15248380251372157] Parent S. Vaillancourt-Morel M.-P. Gillard A. (2022). Interpersonal violence (IV) in sport and mental health outcomes in teenagers. Journal of Sport and Social Issues, 46(4), 323–337. 10.1177/01937235211043652

[bibr37-15248380251372157] Peltola M. Kivijärvi A. (2017). Sports and structured leisure as sites of victimization for children and young people in Finland: Looking at the significance of gender and ethnicity. International Review for the Sociology of Sport, 52(8), 955–971. 10.1177/1012690216636607

[bibr38-15248380251372157] Sanderson J. Weathers M. R. (2020). Snapchat and child sexual abuse in sport: Protecting child athletes in the social media age. Sport Management Review, 23(1), 81–94. 10.1016/j.smr.2019.04.006

[bibr39-15248380251372157] Sølvberg N. Torstveit M. K. Rosenvinge J. H. Pettersen G. Sundgot-Borgen J. (2022). Sexual harassment and abuse among young elite athletes, recreational athletes, and reference students: A prospective study. Medicine & Science in Sports & Exercise, 54(11), 1869–1878. 10.1249/MSS.0000000000002972 PMC9584052 35763597

[bibr40-15248380251372157] Stafford A. Alexander K. Fry D. (2013). Playing through pain: Children and young people’s experiences of physical aggression and violence in sport. Child Abuse Review, 22(4), 287–299. 10.1002/car.2289

[bibr41-15248380251372157] Stafford A. Alexander K. Fry D. (2015). “There was something that wasn’t right because that was the only place I ever got treated like that”: Children and young people’s experiences of emotional harm in sport. Childhood, 22(1), 121–137. 10.1177/0907568213505625

[bibr42-15248380251372157] Stirling A. E. (2009). Definition and constituents of maltreatment in sport: Establishing a conceptual framework for research practitioners. British Journal of Sports Medicine, 43(14), 1091–1099. 10.1136/bjsm.2008.051433 19028734

[bibr43-15248380251372157] Thoma B. C. Rezeppa T. L. Choukas-Bradley S. Salk R. H. Marshal M. P. (2021). Disparities in childhood abuse between transgender and cisgender adolescents. Pediatrics, 148(2), e2020016907. 10.1542/peds.2020-016907 PMC8344346 34226247

[bibr44-15248380251372157] Timon C. E. Dallam S. J. Hamilton M. A. Liu E. Kang J. S. Ortiz A. J. Gelles R. J. (2022). Child sexual abuse of elite athletes: Prevalence, perceptions, and mental health. Journal of Child Sexual Abuse, 31(6), 672–691. 10.1080/10538712.2022.2100026 35821644

[bibr45-15248380251372157] Tsuchiya S. Tsuchiya M. Momma H. Sekiguchi T. Kuroki K. Kanazawa K. Koseki T. Igarashi K. Nagatomi R. Hagiwara Y. (2017). Factors associated with sports-related dental injuries among young athletes: A cross-sectional study in Miyagi prefecture. BMC Oral Health, 17(1), 168. 10.1186/s12903-017-0466-2 PMC5747271 29284466

[bibr46-15248380251372157] Tuakli-Wosornu Y. A. Burrows K. Fasting K. Hartill M. Hodge K. Kaufman K. Kavanagh E. Kirby S. L. MacLeod J. G. Mountjoy M. Parent S. Tak M. Vertommen T. Rhind D. J. A. (2024). IOC consensus statement: Interpersonal violence and safeguarding in sport. British Journal of Sports Medicine, 58(22), 1322–1344. 10.1136/bjsports-2024-108766 39586634

[bibr47-15248380251372157] Vertommen T. Decuyper M. Parent S. Pankowiak A. Woessner M. N. (2022). Interpersonal violence in Belgian sport today: Young athletes report. International Journal of Environmental Research and Public Health, 19(18), Article 18. 10.3390/ijerph191811745 PMC9517528 36142018

[bibr48-15248380251372157] Vertommen T. Kampen J. Schipper-van Veldhoven N. Wouters K. Uzieblo K. Van Den Eede F. (2017). Profiling perpetrators of interpersonal violence against children in sport based on a victim survey. Child Abuse & Neglect, 63, 172–182. 10.1016/j.chiabu.2016.11.029 27923185

[bibr49-15248380251372157] Vertommen T. Schipper-van Veldhoven N. H. Hartill M. J. Van Den Eede F. (2015). Sexual harassment and abuse in sport: The NOC*NSF helpline. International Review for the Sociology of Sport, 50(7), 822–839. 10.1177/1012690213498079

[bibr50-15248380251372157] Vertommen T. Schipper-van Veldhoven N. Wouters K. Kampen J. K. Brackenridge C. H. Rhind D. J. A. Neels K. Van Den Eede F. (2016). Interpersonal violence against children in sport in the Netherlands and Belgium. Child Abuse & Neglect, 51, 223–236. 10.1016/j.chiabu.2015.10.006 26516053

[bibr51-15248380251372157] von Elm E. Altman D. G. Egger M. Pocock S. J. Gøtzsche P. C. Vandenbroucke J. P. (2007). The strengthening the reporting of observational studies in epidemiology (STROBE) statement: Guidelines for reporting observational studies. The Lancet, 370(9596), 1453–1457. 10.1016/S0140-6736(07)61602-X 18064739

[bibr52-15248380251372157] Ward L. Strashin J. (2019, February 10). Sex offences against minors: Investigation reveals more than 200 Canadian coaches convicted in last 20 years | CBC Sports. https://www.cbc.ca/sports/amateur-sports-coaches-sexual-offences-minors-1.5006609

[bibr53-15248380251372157] Wilinsky C. L. McCabe A. (2021). A review of emotional and sexual abuse of elite child athletes by their coaches. Sports Coaching Review, 10(1), 84–109. 10.1080/21640629.2020.1775378

[bibr54-15248380251372157] Woessner M. N. Pankowiak A. Kavanagh E. Parent S. Vertommen T. Eime R. Spaaij R. Harvey J. Parker A. G. (2024). Telling adults about it: Children’s experience of disclosing interpersonal violence in community sport. Sport in Society, 27(5), 661–680. 10.1080/17430437.2023.2268555

[bibr55-15248380251372157] World Health Organization. (2022, September 19). Child maltreatment. https://www.who.int/news-room/fact-sheets/detail/child-maltreatment

