# Supplementary material for: Gender Differences in Sports-Related Child Maltreatment: A Systematic Review
Source: Trauma Violence Abuse. 2025 Sep 27;27(5):2110–25. doi: 10.1177/15248380251372157 (PMC13736285; doi:10.1177/15248380251372157)
Supplement: Supplementary file 1 [file sj-docx-1-tva-10.1177_15248380251372157.docx]

**Supplemental Table 1**

*Quality Assessment of Included Articles using the STROBE Checklist*

| Study | STROBE Rating |
| --- | --- |
| Boudreault et al., 2022 | 19 |
| Brackenridge et al., 2008 | 19 |
| Daignault et al., 2023 | 20 |
| Hartill & Lang, 2018 | 15 |
| Hartill et al., 2023 | 22 |
| Nielsen, 2001 | 15 |
| Pankowiak et al., 2023 | 21 |
| Parent et al., 2016 | 21 |
| Parent & Vaillancourt-Morel, 2021 | 19 |
| Parent et al., 2022 | 18 |
| Peltota & Kivijärvi, 2017 | 17 |
| Sanderson & Weathers, 2020 | 18 |
| Sølvberg et al., 2022 | 21 |
| Stafford et al., 2013 | 18 |
| Stafford et al., 2015 | 19 |
| Timon et al., 2022 | 21 |
| Tsuchiya et al., 2022 | 19 |
| Vertommen et al., 2022 | 21 |
| Vertommen et al., 2017 | 21 |
| Vertommen et al., 2015 | 18 |
| Vertommen et al., 2016 | 20 |
| Woessner et al., 2024 | 20 |
